# Dispersion-Engineered Surface Phonon Polariton Metasurfaces for Tunable and Efficient Polarization Conversion

**DOI:** 10.1021/acs.nanolett.5c02708

**Published:** 2025-08-11

**Authors:** Raghunandan B. Iyer, Sang Hyun Park, Ramachandra Bangari, S. Maryam Vaghefi Esfidani, Tony Low, Thomas G. Folland

**Affiliations:** † Department of Physics and Astronomy, 4083 The University of Iowa, Iowa City, Iowa 52245 United States; ‡ Department of Electrical and Computer Engineering, 5635 University of Minnesota, Minneapolis, Minnesota 55455, United States

**Keywords:** Surface Phonon Polaritons (SPhPs), Metasurfaces, Polarization Conversion, Mid-Infrared Photonics, Dispersion Engineering, Tunable Optical Devices

## Abstract

Metasurfaces offer compact control of light polarization, which is vital for imaging, sensing, and communications. However, cost-effective efficient polarization conversion in the mid-infrared (IR) remains challenging due to reliance on high-resolution lithography. We demonstrate that dispersion engineering of surface phonon polariton (SPhP) metasurfaces overcomes these limitations, enabling efficient and tunable polarization conversion in the mid-IR. By integrating a dielectric layer with the SiC-based metasurface, we control hybrid SPhP and SPhP-like waveguide resonances, achieving up to 61% experimental and 82% simulated conversion efficiency across the Reststrahlen band. Our design avoids SiC etching, enhancing compatibility with hard-to-etch materials. Bandwidth tunability is achieved by varying the grating pitch, with full width at half-maximum ranging from 146.82 to 52.2 cm^–1^ (∼15% to 5% of design frequency), enabling versatile applications ranging from broadband spectroscopy to narrow band thermal sensing. This platform is transferable to other SPhP materials, offering new avenues for reflective polarizers in the mid-IR and terahertz ranges.

## Graphical abstract

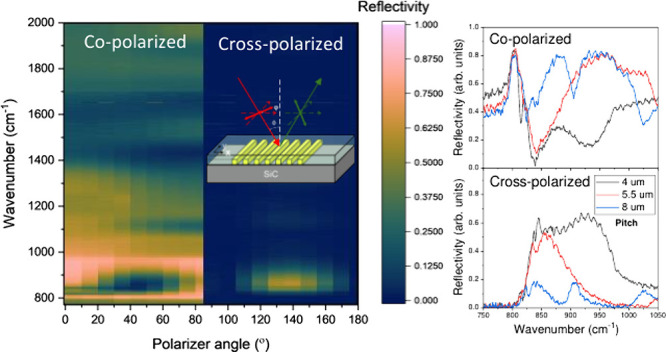

Efficient polarization control is pivotal for advancing optical devices across diverse applications, including sensing,^1^ spectroscopy,^2,3^ imaging,^4^ communication,^5^ thermal management,^6^ and nonlinear optical devices.^7^ Although polarization conversion can be realized in optical systems utilizing anisotropic birefringent materials,^8–10^ the chalcogenide single-crystal materials generally used in the infrared are bulky and expensive. As such, the design flexibility of metasurfaces—composed of subdiffractional structures on a surface—has enabled the realization of a wide range of polarization-controlling metasurfaces.^11–16^ Despite this progress, realizing general-purpose high-efficiency polarization converters that offer tunability between both broadband^17^ and narrowband^18^ remains a significant challenge, particularly in the mid-infrared (mid-IR) regime.^19,20^ This can partly be attributed to the relatively limited control one can achieve over metasurface antennas fabricated by using conventional metals or dielectrics alone. Furthermore, complex metasurface designs, such as nanoantennas, chiral nanostructures, and coupled planar structures, involve intensive electron-beam lithography processes and often result in higher optical losses owing to the use of metals.^21^

In this work, we demonstrate a dispersion-engineered surface phonon polariton (SPhP) metasurface that overcomes these limitations, enabling bandwidth-tunable polarization conversion with high efficiency in the mid-IR. Mode dispersion engineering in metasurfaces relies on manipulating the geometry and materials within a structure to tune the velocity of surface waves and control properties of light including phase,^22^ amplitude,^23^ polarization,^24,25^ aberration,^26,27^ focal lengths,^28^ etc. SPhPs are evanescent modes that arise from the resonant coupling of infrared light with optical phonons at the surface of polar dielectric materials between the transverse optical (TO) and longitudinal optical (LO) phonon frequencies, which are termed the Reststrahlen band. They exhibit strong field confinement and long lifetimes in the mid-infrared spectral range, making them highly attractive for applications in sensing, thermal emission control, and subwavelength optics. However, SPhP-based nanophotonic systems are often inherently narrowband, which traditionally limits their applications outside narrowband spectroscopy. Our work builds upon the foundation of SPhP-like waveguide (WG) modes discussed by Passler et al.,^29^ which used prism-based spectroscopy to demonstrate the existence of these modes. Our work shows how these modes interact with substrate SPhPs and SPPs in a metasurface structure. This enables emergent phenomena such as tunable dichroism and polarization conversion not explored in prior studies and relevant for far-field device orientation. Moreover, with this approach, we can use simple periodic metallic gratings with a dispersion-tailored dielectric overlayer fabricated using photolithographically realizable feature sizes to engineer hybrid modes that achieve high polarization conversion efficiency. This strategy provides a fundamentally different approach to engineering polarization in metamaterials compared to conventional antennas, enabling tunable and highly efficient polarization conversion with reduced fabrication complexity.

Presently, we showcase tailoring the SPhP resonances in the Reststrahlen band of a silicon carbide (SiC)-based metasurface since the polar material allows for efficient control of resonances in the mid-IR region.^30–34^ SiC metasurfaces are relevant to applications in coherent thermal sources,^35–37^ vibrational mode control,^38^ and quantum information sciences,^39^ thereby broadening the scope of the approach. The metasurface structure consists of a periodic gold array of subwavelength features on a SiC substrate to leverage the SPhP modes, which are further modified by a dielectric coating on the surface. By adjusting and varying the grating pitch of the metasurface, we couple the material to the correct position on the SPhP dispersion, enabling efficient and tunable polarization conversion. A key feature of our approach lies in the interplay between SPhPs in the substrate, the SPPs in gold strips, and the modes in the dielectric layer on the metasurface, which together enhance the light–phonon interaction, thereby amplifying both dichroism and polarization conversion. Furthermore, unlike conventional approaches for metasurfaces that rely on etching SiC,^36,38,40–42^ we achieve this functionality without etching, paving the way for broader applicability in phonon polariton materials that are challenging to etch.^43–46^

Figure 1a provides a schematic overview of the metasurface and the optical measurement setup designed to investigate the interaction between photonic and phononic modes.^47^ The metasurface consists of a SiC substrate with a lithographically fabricated gold grating and an amorphous silicon (a-Si) dielectric layer on top. The samples were characterized by Fourier-transform infrared (FTIR) microscopy, employing a polarizer–analyzer configuration, following the design in ref 47. Co-polarized measurements, where the polarizer and analyzer are aligned parallel and cross-polarized measurements are set orthogonally, enable detailed analysis of dichroism and polarization-dependent behavior in the metasurface. The polarizer angle relative to the grating vector determines the azimuthal orientation of the electric field on the metasurface. We used a 25× Cassegrain objective (Thorlabs LMM25XF-P01) with a numerical aperture of 0.4° and an average angle of ∼20°. Measurements are presented between 700 cm^–1^ and 2000 cm^–1^ to capture the spectral signatures of SPhPs between 800 cm^–1^ and 970 cm^–1^ and dielectric resonances between 1000 cm^–1^ to 1800 cm^–1^, offering a comprehensive understanding of the metasurface’s dichroic properties.

**Figure 1:**
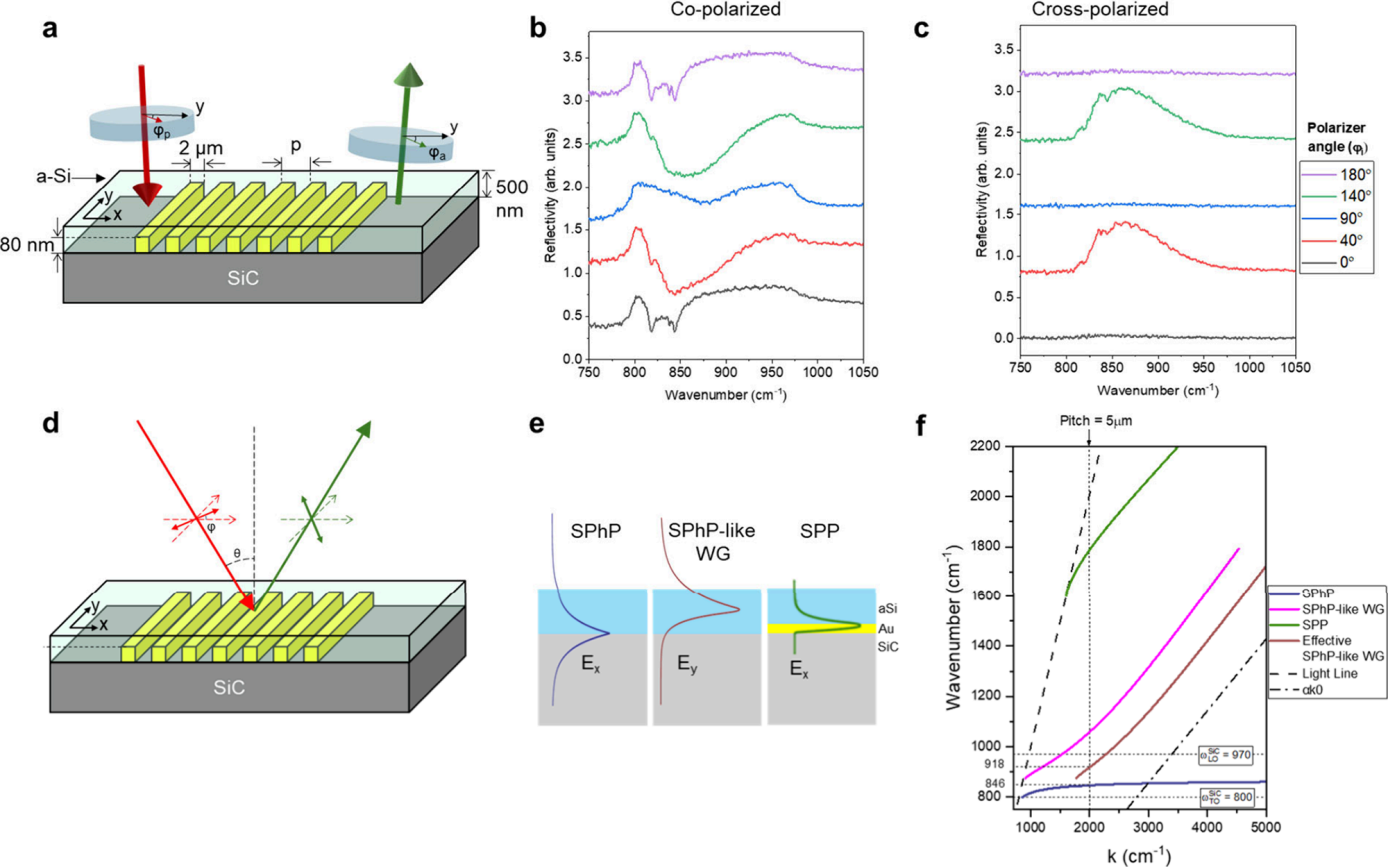
Overview of the phononic metasurface and optical characterization. (a) Schematic of the metasurface, consisting of a SiC substrate with a gold grating and a-Si dielectric layer, (b) spectral plots of reflectivity at selected polarizer angles (0°, 40°, 90°, 140°, 180°) in co-polarized configuration, (c) spectral plots of reflectivity in cross-polarized configuration corresponding to polarizer angles shown in co-polarized configuration spectra, (d) schematic of polarization rotation/conversion in the metasurface at intermediate angles, (e) schematic of field profiles of SPhP and SPhP-like WG modes at the SiC/aSi-dielectric interface and SPP modes in the gold/aSi–dielectric interface, and (f) calculated dispersion diagram of SPhPs, SPPs, SPhP-like WG, and effective SPhP-like WG modes shown in (e).

Figure 1b illustrates the polarization-dependent response of the metasurface within the Reststrahlen band, presented as reflectivity spectra at selected polarizer and analyzer angles with respect to the grating vector: 0°, 40°, 90°, 140°, and 180°. The metasurface under investigation features a grating with a pitch of 5 μm, specifically designed to tune the dispersion and interaction of various modes. The grating provides the necessary momentum for the resonant excitation of SPhPs within the Reststrahlen band of the SiC metasurface. Key resonant features are observed at 818.9 cm^–1^, 838 cm^–1^, and 843.6 cm^–1^, with corresponding *Q*-factors of 83.3, 374.6, and 208.3, respectively. The dips at 818.9 cm^–1^ and 843.6 cm^–1^ are blue-shifted SiC SPhP modes traveling in opposite directions. The high *Q*-factors of the modes indicate strong energy confinement and low radiative losses. Furthermore, adjusting the polarizer angle modulates the orientation of the electrical field along the grating vector, thereby tuning the position and intensity of these resonance dips. As such, this approach reveals a strong dependence of the modes on the polarization angle, which offers a degree of tunability to the phonon polariton resonances.

We examined the metasurface’s ability to control the polarization of light by using a cross-polarized configuration. Figure 1c illustrates the cross-polarized response corresponding to the spectra presented in Figure 1b. The data, normalized against gold spectra in the co-polarization configuration, reveal an enhanced cross-polarized reflectivity response with a maximum polarization conversion efficiency (PCE) of 61% at 864.4 cm^–1^, indicating a highly efficient rotation of the reflected light’s polarization at an azimuthal angle of 40°. Even though this effect can be observed outside the Reststrahlen band of SiC, it is much weaker, as will be discussed later. The schematic shown in Figure 1d illustrates the polarization rotation due to the interaction with the metasurface at intermediate angles. At a pitch of 5 μm, the bandwidth of the polarization conversion spans about a full-width at half-maximum (fwhm) of 82.69 cm^–1^ (∼50% of the Reststrahlen band of SiC).

Figure 1e illustrates the various optical modes supported by the metasurface at different material interfaces. An SPhP mode propagates along the surface of the polar crystal (i.e., SiC). It exhibits a dominant in-plane *E*_*x*_ field component, which peaks at the SiC/aSi-dielectric interface, as indicated by the blue curve. The corresponding dispersion relation for this SPhP mode is plotted as a blue line in Figure 1f. Additionally, an in-plane *E*_*y*_ electric field profile (red line in Figure 1e) is associated with the guided mode at the air/aSi-dielectric/SiC interface, exhibiting stronger confinement compared to a mode bound solely by air.^29^ The dispersion of this air/aSi-dielectric/SiC-bound waveguide mode, shown by the red line in Figure 1f, asymptotically approaches the light line of the dielectric (*c*/*n*_dielectric_) at large wavevectors. However, at smaller momenta, the dispersion bends upward due to the influence of the SiC substrate, resembling the SPhP dispersion.^29^ Notably, this SPhP-like WG mode is transverse electric (TE) polarized (excited by s-polarized light), distinguishing it from the transverse magnetic (TM) SPhP modes (excited by p-polarized light). Furthermore, the SPPs supported at the Au/aSi–dielectric interface are represented by the green line in Figures 1e,f. These TM SPPs feature an in-plane electric field tightly confined at the metal–dielectric boundary, with a dispersion that lies very close to the light line in the dielectric (∼*k*_0_) particularly in the Reststrahlen band of SiC. Since the metasurface structure periodically alternates between SiC/aSi-dielectric and SiC/Au/aSi-dielectric regions, we calculated a correction to account for the effective optical mode of the periodic system. The corrected dispersion of the effective SPhP-like WG mode, indicated by the maroon line in Figure 1f, was obtained using

$$(1-FF)\cdot k_{WG}+FF\cdot \alpha \cdot k_{0}=\frac{2\pi }{\Lambda }$$ 1

where FF is the fill fraction defined as the ratio of the grating width (*d*) to the pitch (Λ). The correction factor α was determined empirically, as discussed later. For the metasurface analyzed here, with a grating width of 2 μm and a pitch of 5 μm (FF = 0.4), α was found to be approximately 4, a value consistent with the refractive index of the dielectric layer. This correction (maroon line) shows that the effective SPhP-like WG mode shifts to higher momenta than the uncoupled modes. Figure 1f also overlays the Reststrahlen band of SiC and the momentum line corresponding to a grating pitch of 5 μm. At this pitch, incident light couples into a combination of metasurface modes, namely, the SPhP (around 846 cm^–1^) and the effective SPhP-like WG mode (extending up to ∼948 cm^–1^). The dielectric-guided modes’ steep dispersion contributes to the metasurface’s broadband optical response. Importantly, the observed polarization rotation results from the effective coupling between the *E*_*x*_ field of SPhP and the *E*_*y*_ field of the hybridized waveguide mode in the metasurface. We have also experimentally demonstrated the role of dielectrics using a lower index ZnO dielectric layer (Figure S5 in the SI).

To better understand the properties of the structure, we conducted full-wave numerical simulations using the CST Studio Suite. The simulations were carried out with TM-polarized light incident on the grating at an angle of 20° to mirror the co-polarized experimental setup. The optical constants for the dielectric in the simulation were obtained by performing FTIR measurements on large-area dielectric thin films (Figure S6 in the SI). Figure 2a compares the experimental contour plot of reflectivity across various polarizer angles in a co-polarized configuration (shown from 0 to 90°). These are directly compared with results from finite element method (FEM) simulations (from 90 to 180 deg). The results for 0–90° and 90–180° are fully symmetrical and omitted for brevity. The experimental contour plot reveals a sharp yet tunable optical response across the different polarization angles. In Figure 2a, the sharp dark bands at smaller polarization angles (e.g., 0–30°) correspond to highly confined SPhP resonances, evidenced by their sharp spectral signatures within the Reststrahlen band, which was also observed in Figure 1b as high *Q*-factor dips. As the polarization angle increases to the intermediate range (40–70°), a second broad and dark band appears in the co-polarized measurement, suggesting birefringence. At 90°, the geometry prohibits the launching of SPhP modes, but we still observe a broad resonance, suggesting a TE WG mode. This behavior underscores the role that dispersion engineering plays in polarization control, as described earlier. The simulation results closely replicate the key features observed experimentally within the Reststrahlen band. Notably, at higher wavenumbers (1400 cm^–1^ and 1800 cm^–1^), the simulations highlight dielectric waveguiding modes, distinct from the SPhP resonances observed in the Reststrahlen band. Similarly, Figure 2b compares the experimental and simulated contour plots from cross-polarized configuration measurements. The experimental quadrant demonstrates and visualizes the polarization conversion. The simulation quadrant exhibits features similar to those of the experimental results, showing a strong polarization conversion response within the Reststrahlen band. We note a fwhm of 107.9 cm^–1^ (∼62% of the Reststrahlen band) and a maximum PCE of 82.2% at 907.75 cm^–1^ in the simulation results at a 40° azimuthal angle.

**Figure 2:**
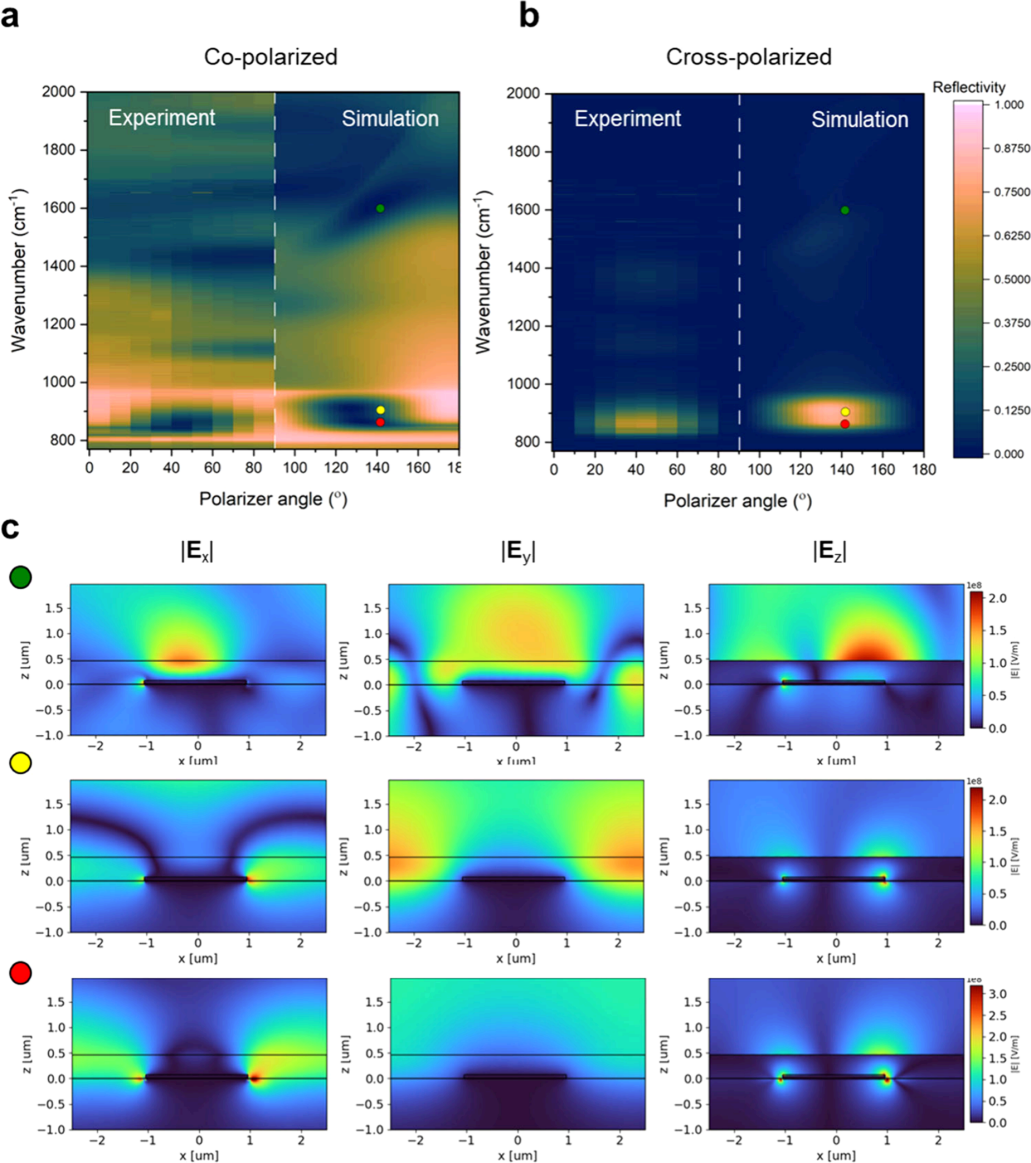
Surface polariton tuning in co-polarized configuration. (a) Left quadrant: Contour plot of reflectivity as a function of polarizer angle, showing polarization-dependent resonances within the Reststrahlen band. Right quadrant: Simulated contour plot of reflectivity under s-polarized light. (b) Left quadrant: Reflectivity contour under cross-polarization showing strong conversion within the Reststrahlen band. Right quadrant: FEM simulations replicating experimental polarization conversion results. (c) Field maps at 40° incidence angle at specific wavenumbers highlighting spatial confinement: 867 cm^–1^ (red dot), 907 cm^–1^ (yellow dot), and 1598 cm^–1^ (green dot).

The field maps in Figure 2c provide a spatial visualization of electromagnetic field confinement at specific wavenumbers along the *x*, *y*, and *z* directions. At 867 cm^–1^ (red dot), near the low-frequency edge of the Reststrahlen band, the *E*_*x*_ and *E*_*z*_ fields are strongly confined at the SiC/aSi interface, while the *E*_*y*_ fields are absent. This localization aligns with the expected field distribution of TM-polarized SPhPs, which exhibit a notable coupling to out-of-plane field components. At 907 cm^–1^ (yellow dot), within the middle of the Reststrahlen band, both *E*_*x*_ and *E*_*y*_ fields are concentrated in the aSi dielectric layer, with a significant component above the structure, characteristic of a dielectric waveguide mode.^29^ The simultaneous presence of *E*_*x*_ and *E*_*y*_ fields suggests polarization conversion effects enabled by TE SPhP-like WG modes. Additionally, the *E*_*x*_ and *E*_*z*_ fields observed at the grating edges highlight the contribution of TM-polarized SPPs as discussed earlier. At 1598 cm^–1^ (green dot), at a significantly higher wavenumber, the fields shift toward the aSi/air interface, signifying a transition to purely dielectric WG modes. However, the field intensities are notably weaker compared with those observed within the Reststrahlen band, underscoring the role of SPhP interactions in enhancing polarization conversion. The mechanism by which resonances with different polarization selection conditions give rise to mode conversion can be explained by considering an anisotropic Fabry–Perot cavity, as discussed in the SI. In brief, a resonator structure with spectrally detuned resonances aligned along separate optical axes exhibits strong polarization conversion when light illuminates the sample at an intermediate angle. This can be attributed to the effective permittivity of a material when light is incident in this orientation.

To better parametrize the tunability of the system, Figure 3 highlights the grating pitch dependence of optical response of the metasurface. The reflectivity measurements were conducted with grating pitches varying from 3.5 to 9 μm with the grating width constant at 2 μm (effectively tuning FF), as shown in Figure 1a, to avoid tuning localized SPPs, enabling a clear distinction between localized and delocalized (propagating) modes that are sensitive to pitch. In the co-polarization configuration (Figure 3a), strong tuning of SPhP modes within the Reststrahlen band is evident, with distinct resonances observed at grating pitches of 4 μm, 5.5 μm, and 8 μm. The cross-polarization spectra (Figure 3b) illustrate a notable narrowing of the polarization conversion bandwidth as the pitch increases, as enabled by dispersion engineered SPhP interactions. This behavior, further visualized in the experimental contour plot of Figure 3b and confirmed by the simulated contour plots in Figure 3c, underscores the pitch-dependent nature of polarization control. By adjusting the pitch of the metasurface, we demonstrated a tunable bandwidth with an fwhm of 146.82 cm^–1^ at a 4 μm pitch and a narrow fwhm of 52.2 cm^–1^ at a 7 μm pitch. The overlay in Figure 3c was derived from the theoretical dispersion of SPhP modes and SPhP-like WG modes by converting the momentum of the mode dispersion to an equivalent grating pitch.

**Figure 3:**
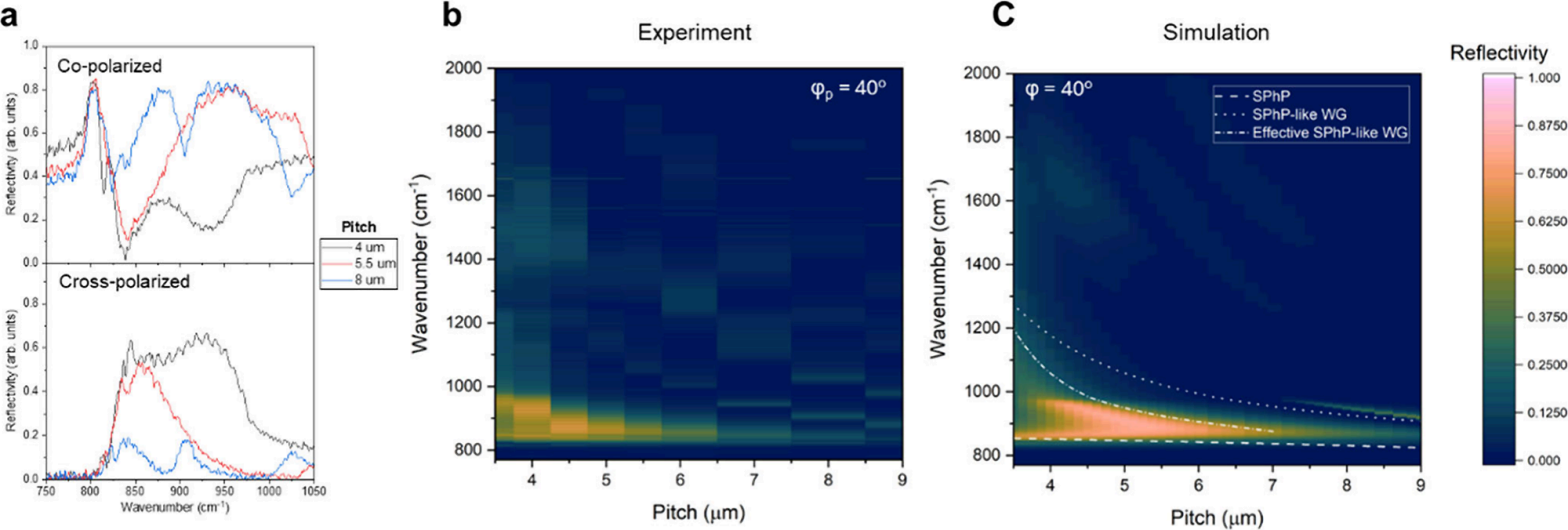
Grating pitch-dependent optical tuning. (a) Reflectivity spectra showing tunable phonon polariton resonances and polarization conversion bandwidth with pitch. (b) Experimental pitch dependence reflectivity contour plots in cross-polarized mode. (c) Simulated pitch dependence reflectivity contour plots in cross-polarized mode. Overlay: Theoretical dispersion of SPhP modes and SPhP-like WG and effective SPhP-like WG modes with the momentum of mode dispersion converted into an equivalent grating pitch.

For the effective SPhP-like WG modes, a modified form of eq 1 was used to incorporate the influence of SPPs through the correction factor α. As mentioned earlier, this factor was determined empirically by adjusting its value to achieve the best overlap between the calculated dispersion curves and the polarization conversion features across different grating pitches, as shown in Figure 3c. A value of α ≈ 4 provided the most consistent alignment with the observed mode boundaries, which is physically reasonable given the refractive index of the dielectric. Figure 3c indicates that the SPhP-like WG mode determines the upper bound of the polarization conversion phenomenon, while the SPhPs set the lower bound. Furthermore, it is worth noting that the Au grating plays a key role in the dispersion engineering of the waveguide mode. The bandwidth at higher grating pitches can be further tuned by increasing the fill factor (FF) of the grating (Figure S2 in the SI), thereby further confirming the role of SPPs in broadening the polarization conversion response. The nondominant modes seen in our results with higher pitches can be attributed to coupling to higher-order diffraction modes, which can be tuned by FF and the optical constants of the dielectric layer (Figure S4 in the SI). The demonstrated tunability of the bandwidth of polarization conversion effects could be key for versatile applications: broad bandwidth features are ideal for spectroscopy, while narrow bandwidth regions are better suited for precise thermal emission.

Figure 4 presents the simulation results at various incidence angles on a 5 μm grating with a constant 40° azimuthal angle. The figure demonstrates that broadband polarization conversion remains consistent from normal incidence to angles exceeding 65°. This indicates the metasurface’s robustness in response over a wide range of incidence angles. The inset in Figure 4 shows normalized cross-polarization experimental results obtained using a ZnSe lens at ∼5° incidence angle and a 25× objective at ∼20° incidence angle, confirming the simulation results. Such wide-angle robustness enables optical components to maintain their performance across a larger field of view, thereby reducing aberrations and minimizing the need for complex lens systems. This characteristic is particularly beneficial for imaging sensing systems, where a broader field of view can capture more information without distortion.

**Figure 4:**
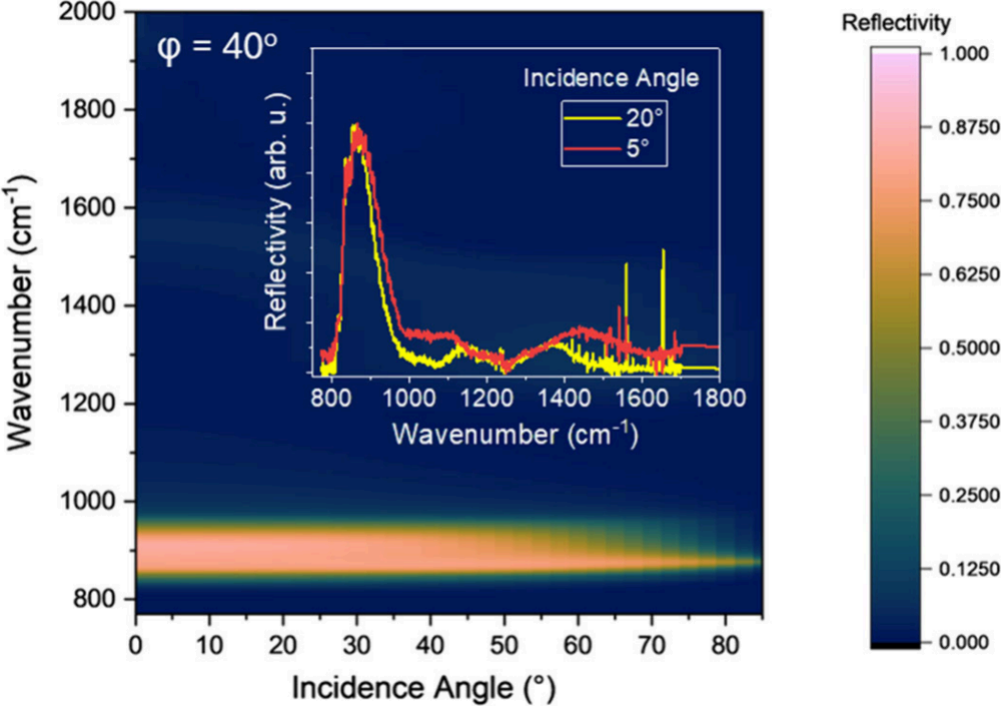
Simulated polarization conversion efficiency across various incidence angles for a 5 μm grating at a fixed 40° azimuthal angle, demonstrating broadband and wide-angle robustness. Inset: Experimental cross-polarization results at ∼5° and ∼20° incidence angles validating the simulations.

We note that our design offers simplicity when compared to recent inverse design studies, which often involve high performance but are structurally complex geometries that can suffer significant performance degradation upon fabrication especially for large-area, volumetric, or freeform implementations in addition to requiring large amounts of computational resources.^48–52^ However, with the introduction of additional structural complexity through targeted design enhancements, the performance of our device can be further enhanced.

In this study, we demonstrate tunable dichroism and polarization conversion in dispersion-engineered metasurfaces through the precise control of hybrid polariton resonances, showcasing the potential for advanced optical manipulation. By modifying the dispersion of SPhPs in SiC-based metasurfaces by interaction with the dielectric modes of an a-Si dielectric layer, we achieved high-efficiency, wide-angle, and simple-geometry polarization conversion in the mid-IR. Our polarization-dependent FTIR microscopy and FEM simulation studies reveal that the polarization conversion is highly efficient and tunable with the pitch of the metasurface: a lower pitch results in narrow bandwidth. In contrast, higher pitch leads to a broad bandwidth. The ability to tune the bandwidth of the polarization conversion effects offers significant advantages for various applications. A broad bandwidth range can benefit spectroscopic techniques, enabling the analysis of a wider range of light spectra. In contrast, a narrow bandwidth is advantageous for thermal sensing applications, allowing more precise measurements of the target analyte bands. Moreover, the findings of this work can be extended to other materials with resonances in different spectral regions of interest, highlighting the versatility and applicability of the approach. This work paves the way for developing versatile metasurfaces with engineered optical functionalities, contributing to advancements in sensing, phonon-engineered optics, and advanced polarization control devices.

## Supplementary Material



## Notes

Comparison of experimental and simulated reflectivity spectra and total E field maps; polarization conversion dependence on grating FF and pitch; simulation of metasurfaces without the dielectric overlayer; simulated metasurfaces with varying ε_dielec_; experimental investigation of metasurfaces with sputtered ZnO dielectric overlayer; dielectric layer FTIR reflectance measurements and dielectric function extraction; sample fabrication methodology; mode conversion from anisotropic resonances (PDF)
