# Supplementary material for: Dispersion-Engineered Surface Phonon Polariton Metasurfaces for Tunable and Efficient Polarization Conversion
Source: Nano Lett. 2025 Aug 11;25(33):12554–61. doi: 10.1021/acs.nanolett.5c02708 (PMC12371866; doi:10.1021/acs.nanolett.5c02708)
Supplement: Supplementary file 1 [file nl5c02708_si_001.pdf]

Dispersion-Engineered Surface Phonon Polariton Metasurfaces for Tunable and Efficient Polarization Conversion

Raghunandan B. Iyer[1], Sang Hyun Park[2], Ramachandra Bangari[1], S. Maryam Vaghefi Esfidani[1], Tony Low[2], and Thomas G. Folland [1*]

[1]Department of Physics and Astronomy, The University of Iowa, Iowa City, Iowa 52245 United States

[2]Department of Electrical and Computer Engineering, University of Minnesota, Minneapolis, Minnesota 55455, United States

*thomas-folland@uiowa.edu

Supplementary Information

1. Comparison of experimental and simulated reflectivity spectra

Figure S1 provides a detailed comparison of the spectra shown in the contour plots of Figure 2a and Figure 2b at polarizer angles of 0°, 40°, and 90°. These plots directly compare the experimental and simulated reflectivities, offering an alternative visualization of the various resonances discussed in the main text. Figure S1c illustrates the absolute values of the total field profiles presented in Figure 2c. The $|\boldsymbol{E}_{\mathrm{total}}|$ profile reveals different modes present at the cross-section of the metasurface at wavenumbers 867, 907, and 1598 cm−1−1. These modes correspond to distinct resonant behaviors within the metasurface, providing deeper insight into the electromagnetic interactions and field distributions at these specific frequencies.

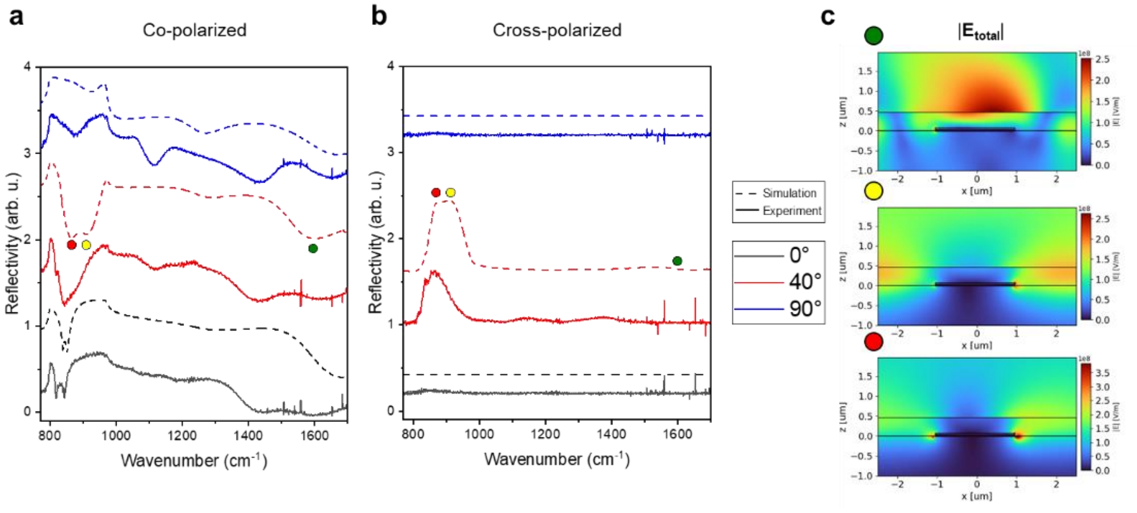

Figure S1. Comparison of experimental and simulated reflectivity spectra at selected polarizer angles (0°, 40°, and 90°), corresponding to the contour plots in Figure 2a,b. (c) Absolute total electric field (|$\mathbf{E}_{\mathbf{total}}$|) profiles at 867, 907, and 1598 cm^{-1}

2. Polarization conversion dependence on grating FF and pitch.

To investigate the role of gold grating parameters in tuning the resonances of the metasurface, we performed numerical simulations by varying the FF of the grating and the results are presented in Figure S2. Figure S2a shows contour plots of the dependence of reflectivity on grating width (or FF) for a constant pitch of 5 µm. We observe a broadening of the BW of polarization conversion as FF increases, demonstrating that the metasurface can be further optimized to control the polarization conversion bandwidth. To further illustrate this effect, we performed additional simulations at FF values of 0.2 and 0.5 for gratings with various pitches, as shown in Figures S2b and S2c. These results indicate that the narrow bandwidths observed in Figures 3b and 3c at higher pitches can be attributed to the lower FF, as those metasurfaces maintained a constant grating width while pitch increased. Furthermore, the results in Figure S2c suggest that the bandwidth at higher pitches can be enhanced by increasing the FF, providing an additional degree of control in designing polarization-selective metasurfaces.

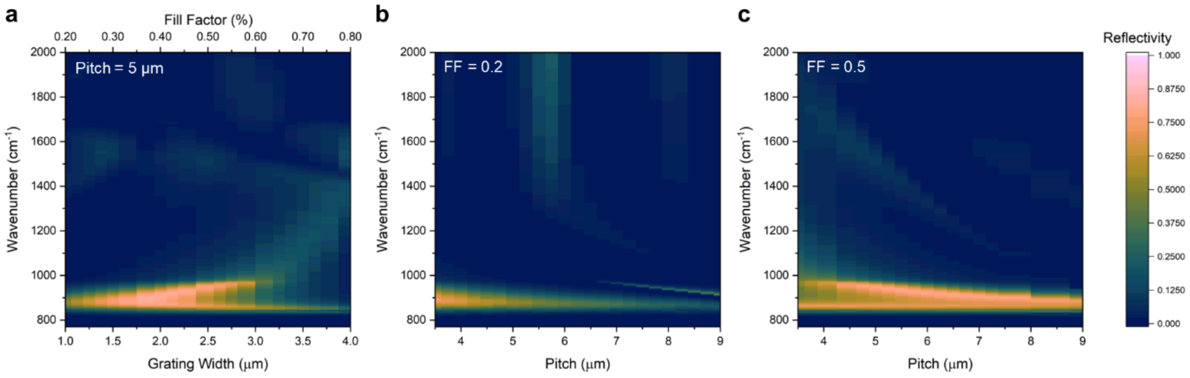

Figure S2. Polarization conversion dependence on grating FF and pitch. (a) Contour plots showing simulated reflectivity as a function of grating width (or FF) at constant 5 µm pitch. (b,c) Reflectivity at FF = 0.2 and 0.5 for various pitches

3. Metasurfaces without dielectric layer

To further investigate the role of the dielectric layer in the metasurface, we performed simulations after removing the dielectric overlayer. The results, presented in Figure S3a, reveal a consistently narrow resonance peak across all FF for a fixed pitch of 5 µm, as well as a similar trend for varying pitches at a constant grating width of 2 µm in figure S3b This persistent peak can be attributed to SPhPs launched in the SiC, facilitated by the grating structure. These findings highlight the crucial role of the dielectric overlayer in broadening the resonance peak through coupled resonances, demonstrating its significance in tailoring the metasurface response.

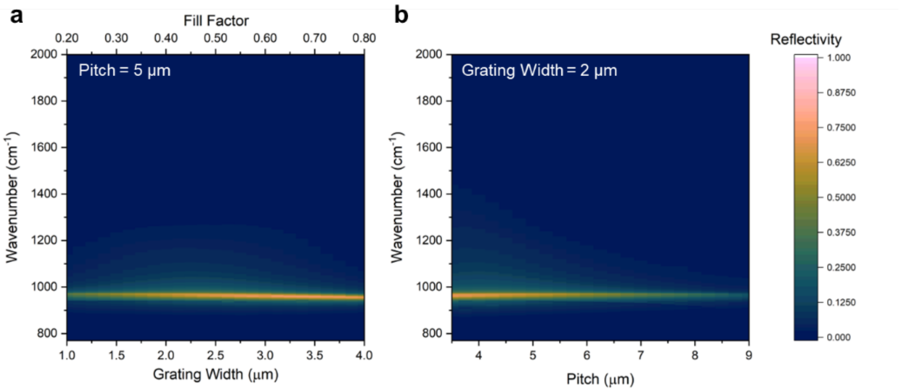

Figure S3. Simulated reflectivity of metasurfaces without the dielectric overlayer. Cross-polarized reflectivity dependence on (a) FF at constant 5 µm pitch and (b) grating pitch with fixed 2 µm grating width

4. Tuning the dielectric layer

To further analyze the influence of the dielectric layer, we performed simulations incorporating the dielectric into the metasurface and examined the dependence of the metasurface response on the dielectric constant (ε_{dielec}) in an idealized lossless scenario. While these simulations may not fully represent physical conditions, they provide valuable insight into the role of the dielectric in shaping the optical characteristics of the metasurface. The co-polarized spectra for varying ε_{dielec} reveal that as ε_{dielec} increases, the SPhP resonances undergo a progressive blue shift due to the increased role of dielectric waveguide modes. As shown in the cross-polarized measurements in Figure S4b, increasing ε_{dielec} facilitates mode hybridization, leading to a broader polarization conversion response. This effect is further visualized in the contour plots of cross-polarization in Figure S4c, where a higher ε_{dielec} results in a significant broadening of the polarization conversion bandwidth.

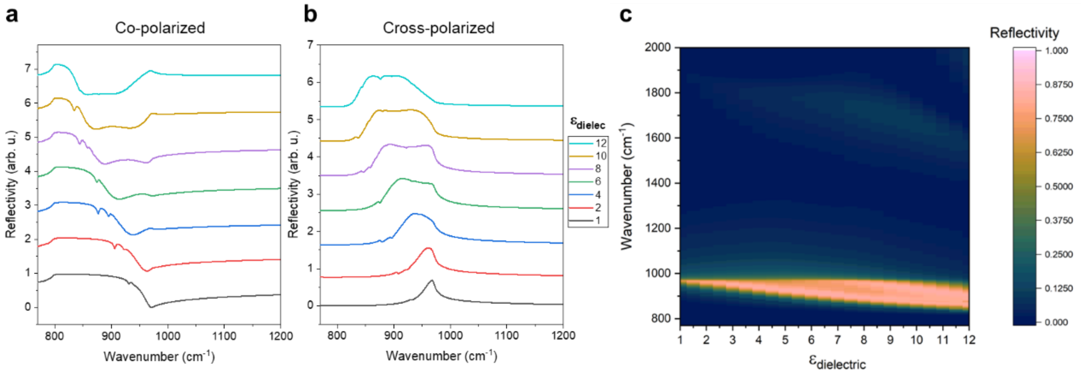

Figure S4. Simulated reflectivity of metasurfaces with varying ε_{dielec} in an idealized lossless case. (a) Co-polarized spectra showing blue shift of SPhP resonances with increasing ε_{dielec} (b) Cross-polarized spectra illustrating enhanced mode hybridization. (c) Contour plots highlighting the broadening of polarization conversion bandwidth with higher ε_{dielec}

5. ZnO dielectric based metasurfaces

To illustrate the tunability of SPhP resonances and polarization conversion, we fabricated metasurfaces using sputtered ZnO as the dielectric layer. Notably, the real part of the permittivity Re(ε) of ZnO is significantly lower than that of a-Si. As shown in the co-polarized spectra in Figure S5a, and in agreement with the simulations in Figure S4a, the SPhP resonances exhibit a redshift and are positioned near the LO edge of the Reststrahlen band. With increasing azimuthal angle, we observe mode hybridization with the dielectric waveguide mode; however, the depth of the resonance dip is constrained by the positioning of the SPhP peaks. Consequently, the polarization conversion bandwidth remains narrow for the metasurface with ZnO, as demonstrated in Figure S5c and as predicted by the simulations in Figure S4c.

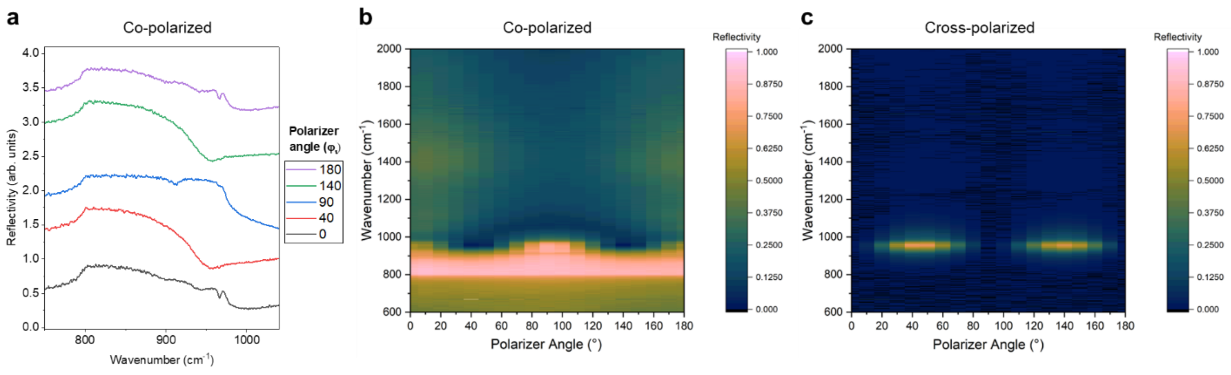

Figure S5. Experimental reflectivity of metasurfaces with sputtered ZnO dielectric layer. (a) Co-polarized spectra showing redshifted SPhP resonances near the LO edge. (b) Co-polarized spectra at selected azimuthal angles illustrating limited mode hybridization. (c) Cross-polarized spectra showcasing narrow polarization conversion bandwidth

6. Dielectric layer Characterization

To characterize the dielectric function of aSi in the mid-IR, we performed FTIR reflectance measurements on aSi thin film deposited on 4H-SiC substrate. We fit the s- and p-polarized reflectance spectra at 20° and 60° incidence (Figure S6a) using the transfer matrix formalism that employs a generalized oscillator model in WVASE ellipsometry software. The dielectric function derived from the fit (Figure S6c) was used in the simulations. Similarly, the dielectric function of Zinc Oxide (ZnO) was extracted from the reflectance of ZnO thin film on gold (Figure S6b).

The oscillator model used for a-Si is expressed as a sum of six resonant terms of the form:

$$\varepsilon(\omega) = \varepsilon_\infty + \sum_{n=1}^{6} \varepsilon_{n\_Gaussian} \qquad (3)$$

with each oscillator being a Gaussian given by:

$$\varepsilon_{n\_Gaussian} = \varepsilon_{n1} + i\varepsilon_{n2}$$

where,

$$\varepsilon_{n2}(\omega) = A_n \exp\left(-\left(\frac{\omega - \omega_n}{\sigma_n}\right)^2\right) - A_n \exp\left(-\left(\frac{\omega + \omega_n}{\sigma_n}\right)^2\right),$$

$$\varepsilon_{n1}(\omega) = \frac{2}{\pi} P \int_0^\infty \frac{\xi \varepsilon_{n2}(\xi)}{\xi^2 - \omega^2} d\xi,$$

$$\sigma_n = \frac{\gamma_n}{2\sqrt{\ln(2)}} \qquad (4)$$

Here, ε_∞ is the high-frequency dielectric constant, A_n is the amplitude, γ_n is the broadening parameter, ω_n is the resonance frequency for the n-th resonance, and ω is the incident frequency. P denotes the Cauchy principal value. The fit parameters are shown in table 1.

The dielectric function of ZnO was modeled using a single TO-LO phonon oscillator of the form:

$$\varepsilon(\omega) = \varepsilon_\infty \frac{\omega_{LO}^2 - \omega^2 - i\gamma_{LO}\omega}{\omega_{TO}^2 - \omega^2 - i\gamma_{TO}\omega} \qquad (5)$$

where ε_∞ is the high-frequency dielectric constant, ω_{TO} and ω_{LO} are the transverse and longitudinal optical phonon energies, γ_{TO} and γ_{LO} are the respective damping factors, and ω is the incident frequency. The fit parameters are shown in table 2.

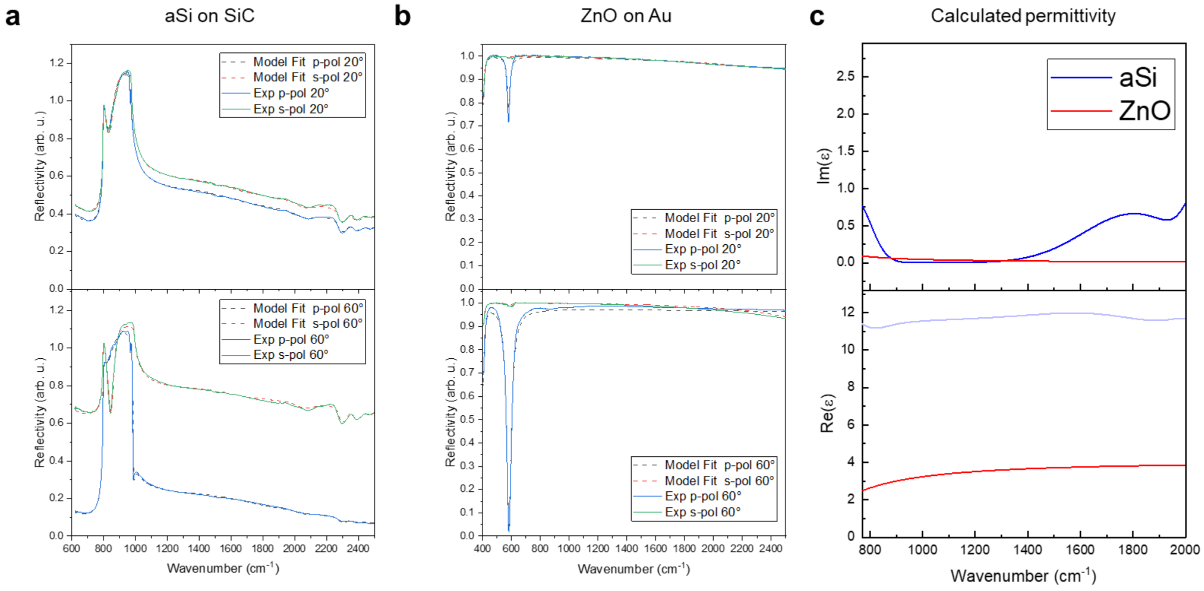

Figure S6. FTIR reflectance measurements and dielectric function extraction for (a) aSi on SiC and (b) ZnO on gold, and (c) Derived dielectric function of a-Si and ZnO used in simulations, obtained via fitting with a generalized oscillator model.

Table 1. Fit parameters for the dielectric function of amorphous silicon (a-Si) in the mid-IR, extracted using a generalized oscillator model with six resonant terms with $\varepsilon_\infty = 11.391$

**Oscillator**	**Amplitude**	**Amplitude Error**	**Energy (1/cm)**	**Energy Error (1/cm)**	**Gamma (1/cm)**	**Gamma Error (1/cm)**
Gauss1	0.7346	0.0201	2057	1.27	124.79	4.11
Gauss2	2.503	0.0624	2306.6	0.366	41.722	0.832
Gauss3	0.99341	0.0234	2391.4	0.792	74.144	2.54
Gauss4	0.29201	0.0201	2472.7	1.4	36.357	3.34
Gauss5	0.6606	0.00927	1803.9	4.8	455.03	10.5
Gauss6	0.84169	0.0137	744.41	1.07	139	1.35

Table 2. Fit parameters for the dielectric function of ZnO in the mid-IR, modeled using a single TO–LO phonon oscillator.

Oscillator	TOLO
ε_∞	4.0233
ε_∞ Error	0.0059
TO Energy (1/cm)	407.28
TO Energy Error (1/cm)	0.0455
TO Gamma (1/cm)	17.675
TO Gamma Error (1/cm)	0.0769
LO Energy (1/cm)	579.08
LO Energy Error (1/cm)	0.0312
LO Gamma (1/cm)	23.247
LO Gamma Error (1/cm)	0.0748

7. Sample Fabrication Methodology:

The metasurface samples were fabricated by embedding a gold grating within a dielectric stack deposited on a SiC substrate, followed by planarization of the top dielectric surface, as illustrated in Figure S7. Initially, an 80 nm thick a-Si layer was deposited on the SiC substrate using plasma-enhanced chemical vapor deposition (PECVD). Standard photolithography (photoresist AZ1512) was then employed to define the grating pattern on the a-Si layer, followed by reactive ion etching (RIE) to etch through the 80 nm a-Si, thereby creating well-defined grating slots. Subsequently, an 80 nm thick gold layer was deposited across the patterned surface. Lift-off was performed to remove the photoresist and excess gold, leaving behind the gold grating embedded within the etched a-Si layer. To achieve a planar top surface and encapsulate the grating structure, an additional 420 nm thick a-Si dielectric layer was deposited over the

entire structure. This fabrication approach ensures a smooth dielectric-air interface while preserving the underlying grating geometry, which is critical for supporting hybrid SPhP and waveguide modes.

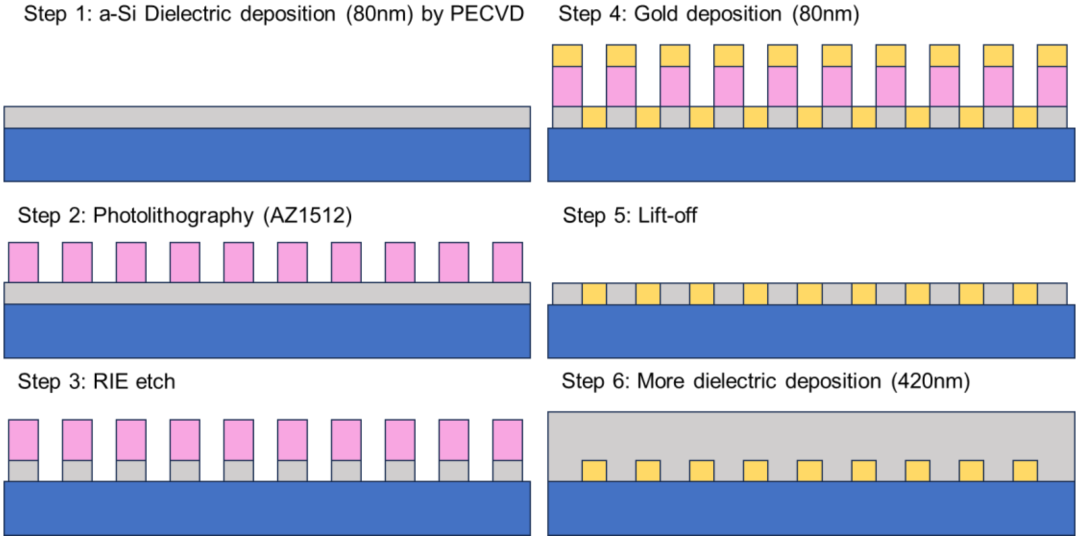

Figure S7. Schematic illustration of the metasurface fabrication process

8. Mode Conversion from anisotropic resonances:

The mechanism of polarization conversion from anisotropic resonances can be understood through an anisotropic Fabry-Perot cavity consisting of a birefringent thin film on a SiC substrate. We consider a birefringent thin film of thickness (d) with a dielectric function of the form;

$$\bar{\bar{\varepsilon}} = \begin{pmatrix} \varepsilon_x & 0 & 0 \\ 0 & \varepsilon_y & 0 \\ 0 & 0 & \varepsilon_y \end{pmatrix} \quad (6)$$

The resonance condition for the Fabry-Perot modes on the mirror-like SiC surface can b expressed as the resonance condition $f = \frac{c}{2\sqrt{\varepsilon}d}(N + 0.5 - \varphi)$, where N is an integer and φ is the phase shift on reflection. At normal incidence, the difference between ε_x and ε_y gives a change in the resonance frequency when comparing the reflection when the electric field is aligned in the E_x and E_y directions. This is simulated in Figure S7a using a multi-layer transfer matrix model described in Nikolai et. al[1]. For a given thickness of the thin film, the resonance frequencies (here 905 and 850cm^{-1}) are controlled by the two permittivity values of the anisotropic dielectric and the thickness of the film. One can vary the thickness of the films while maintaining the same resonance frequencies by adjusting the associated dielectric functions. For thinner films, the dielectric function increases dramatically, and the amount of birefringence (the difference

between dielectric functions) also increases (shown in Figure S7b). We highlight that the values of the dielectric function for the film thicknesses are unphysical and cannot be achieved in a real dielectric but are used to represent a stronger light-matter coupling from a metasurface. We can then consider the reflection of light when the electric field is instead aligned at 45 degrees to the optical axes. In this case, the electric field experiences a modified dielectric function, which can be determined by applying an Euler rotation to the dielectric function of the thin film.

$$\overline{\overline{\varepsilon'}} = \begin{pmatrix} \frac{1}{2}(\varepsilon_x + \varepsilon_y) & \frac{1}{2}(\varepsilon_y - \varepsilon_x) & 0 \\ \frac{1}{2}(\varepsilon_y - \varepsilon_x) & \frac{1}{2}(\varepsilon_x + \varepsilon_y) & 0 \\ 0 & 0 & \varepsilon_y \end{pmatrix} \quad (7)$$

In this case, the resonance condition of the mode occurs at an intermediate frequency, and a large off-diagonal term is present. The result is that there is a considerable conversion to the orthogonal polarization state. This is observed in simulations shown in Figure S7c, with the thinnest films exhibiting ~90% conversion of incident light in the second polarization state. Furthermore, the incident electric field is nearly perfectly absorbed, resulting in a near-perfect polarization state for the film's output. The silicon carbide absorbs the remaining light. These results suggest that surface phonon polariton-based polarization converters, which utilize modes with different polarization selection, can achieve loss-limited performance. For sufficiently low-loss systems, this efficiency could reach 100%.

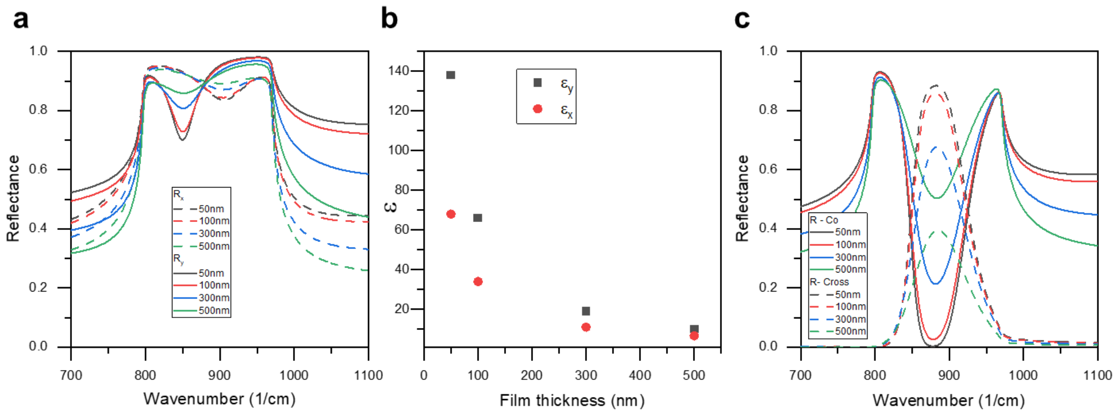

Figure S8. Polarization conversion in anisotropic Fabry-Perot cavities. a) shows the spectral reflectance of a thin film of an anisotropic dielectric on a 4H SiC substrate when the reflectance is polarized along the two principal crystal axes. b) shows the dielectric function used to generate the data in a) as a function of film thickness. c) shows the co- and cross-polarized response of the same films when illuminated with the electric field at 45 degrees to both principal crystal axes.

References

(1) Passler, N. C.; Paarmann, A. Generalized 4 × 4 Matrix Formalism for Light Propagation in Anisotropic Stratified Media: Study of Surface Phonon Polaritons in Polar Dielectric Heterostructures: Erratum. *Journal of the Optical Society of America B* **2019**, *36* (11), 3246. https://doi.org/10.1364/josab.36.003246.
